# Defense and Adaptive Strategies of *Crithmum maritimum* L. Against Insect Herbivory: Evidence of Phenotypic Plasticity

**DOI:** 10.3390/plants14213403

**Published:** 2025-11-06

**Authors:** Liliya Naui, Yassine M’rabet, Bilel Halouani, Najet Chaabene, Faten Mezni, Abdelhamid Khaldi, Karim Hosni

**Affiliations:** 1 National Institute of Agronomy of Tunisia, University of Carthage, Tunis 1082, Tunisia; halouanibilel9@gmail.com; 2 Laboratory of Management and Valorization of Forest Resources, The National Research Institute of Rural Engineering Water, and Forestry, Tunis 2080, Tunisia; faten-mez@hotmail.com (F.M.); khalditn@yahoo.fr (A.K.); 3 National Institute for Research and Physico-Chemical Analysis, Sidi Thabet 2020, Tunisia; yassine.mrabet@gmail.com (Y.M.); najet.chaabene@gmail.com (N.C.); karim.hosni@inrap.rnrt.tn (K.H.)

**Keywords:** *Crithmum maritimum* L., insect herbivory, phenotypic plasticity, resistance–tolerance trade-off, biochemical defense, reproductive allocation

## Abstract

Insect herbivory exerts strong selective pressure on plants, yet no study has documented its effects on the halophytic Apiaceae *Crithmum maritimum* L. (sea fennel). Here, we present the first evidence of natural insect attack on this species, based on five Tunisian coastal populations distributed along a transparent bioclimatic gradient—from sub-humid to semi-arid—and exposed to different levels of herbivory. We implemented an integrative, multi-trait analytical design encompassing morphological, biochemical, mineral, and lipophilic datasets. Each dataset was explored through a suite of complementary multivariate analyses, including ANOVA coupled with Tukey’s HSD, principal component analysis (PCA), partial least squares discriminant analysis (PLS-DA) with variable-importance-in-projection (VIP) scores, correlation matrices, hierarchical clustering, and distance-based redundancy analysis (dbRDA). This integrative strategy provided a robust framework for disentangling the complex trait associations underlying two distinct defense syndromes. Populations from low-herbivory, sub-humid sites (Tabarka, Bizerte, Tunisia) showed higher levels of phenolics, tannins, antioxidants, sterols, PUFA, and structural robustness, indicating a tolerance strategy. Conversely, high-herbivory, semi-arid sites (Haouaria, Monastir, Tunisia) were marked by elevated apiol and terpene levels, sodium and phosphorus accumulation, and reproductive adjustments, reflecting a resistance strategy. The site Cap Negro exhibited a transitional expression, revealing intermediate phenotypic plasticity. These findings show that herbivory intensity and bioclimatic conditions jointly influence the defense syndromes of *C. maritimum*, emphasizing its remarkable phenotypic plasticity and providing the first ecological evidence of insect herbivory in sea fennel.

## 1. Introduction

Insect herbivory is one of the most pervasive biotic stresses affecting plants, with consequences that extend well beyond the direct removal of tissues. Feeding by insects can significantly alter plant growth, reproduction, and survival, shaping ecological interactions and agricultural productivity [1]. A large body of research demonstrates that herbivory induces measurable changes in biochemical and morphological traits, reflecting strategies by which plants mitigate damage and maintain fitness [2].

From a biochemical perspective, insect feeding often triggers the buildup of phenolic compounds, tannins, and other antioxidants. These serve as chemical defenses that reduce how tasty the plant is, hinder insect development, or discourage feeding [3,4]. These compounds are key components of the plant’s defense system and can be quickly mobilized after herbivore attack. Antioxidant tests like 2,2-diphenyl-1-picrylhydrazyl (DPPH) and 2,2′-azino-bis (3-ethylbenzothiazoline-6-sulfonic acid) (ABTS) are commonly used to measure these responses, as they provide overall estimates of radical-scavenging activity and reflect the plant tissues’ ability to fight oxidative stress [5]. For example, a study on the physiological response of wild carrot (*Daucus carota* L.) to the jumping plant louse (*Bactericera trigonica* Ho) showed that DPPH scavenging activity was significantly lower in infested plants and was inversely related to antioxidant enzyme activities [6]. Similarly, research on *Populus tremula* indicated that herbivore damage leads to notable changes in phenolic compounds and antioxidant levels, highlighting the importance of these markers in plant–insect interactions [7].

Tannins are another key group of metabolites that often increase under insect pressure, acting as feeding deterrents or reducing digestibility [8]. The role of these compounds has been well documented in several taxa, including legumes and Fabaceae species, where herbivory induces higher tannin levels and correlates with elevated antioxidant activity [9]. These findings demonstrate that herbivory-driven phenolics, tannins, and antioxidant capacity shifts represent a common defensive strategy across diverse plant lineages.

In addition to biochemical adjustments, insect feeding can also drive morphological changes. These include reductions in stem thickness, fruit or seed size alterations, and decreases in reproductive output that collectively compromise plant fitness [10]. For example, in *Helianthus annuus*, seed-feeding insects have been shown to reduce seed weight and germination capacity significantly [11]. Similarly, damage to developing fruits in Brassica species leads to altered pod morphology and lower seed viability, demonstrating that insect attack can directly impair reproductive success [12]. Structural modifications at the organ level, such as thicker epidermis, changes in leaf area, or altered trichome density, have also been reported as physical defenses against herbivores [13]. These findings highlight the necessity of evaluating both morphological and biochemical responses together, since plants often deploy coordinated strategies that integrate structural and chemical defense mechanisms [14].

Within this framework, halophytes have recently gained attention due to their resilience in extreme environments and rich reservoir of bioactive compounds [15]. Among them, sea fennel (*Crithmum maritimum* L.), a perennial aromatic species of the Apiaceae family, has emerged as a promising candidate for sustainable agriculture and functional food development. Traditionally consumed in Mediterranean regions, it has been recognized for its high content of phenolics, tannins, and essential oils and its potent antioxidant properties [16,17,18]. Recent studies have reported significant antioxidant activity in sea fennel extracts using DPPH and ABTS assays, confirming its potential as a source of natural antioxidants [19]. Furthermore, intraspecific variation in volatile organic compounds and phytochemical composition across Mediterranean populations suggests that *C. maritimum* can adjust its chemical profile to environmental conditions [20].

Despite this growing body of literature on its phytochemistry and nutritional value, one crucial dimension has remained unexplored: the role of biotic stress. To date, no study—or any peer-reviewed work—has reported natural insect herbivory on *C. maritimum*, let alone investigated its effects on morphology and antioxidant markers. Existing studies have focused primarily on abiotic stresses such as salinity, drought, or temperature, but insect–plant interactions in this species remain undocumented [21,22]. The only mention of insect interactions comes from ecological notes reporting seed predation by specialist caterpillars (*Aethes* spp.) in Spain [23]. Still, these observations did not quantify herbivory’s biochemical or morphological consequences.

This gap is significant for two reasons. First, the impact of insect herbivory on bioactive compounds is well established in other species, yet has never been demonstrated in *C. maritimum*. Second, given the economic and ecological importance of sea fennel as an emerging halophyte crop, understanding how it responds to insect attacks is essential for its conservation and domestication.

The present study is therefore the first to report insect herbivory on *C. maritimum*. We investigated five Tunisian coastal populations distributed along a bioclimatic gradient (from sub-humid to semi-arid), assessing whether insect attack was associated with changes in (i) morphological traits, (ii) biochemical markers including total phenolics, tannins, and antioxidant capacity (DPPH, ABTS), (iii) mineral composition, and (iv) lipophilic fraction. We further tested whether herbivory effects interacted with climatic context using integrative statistical approaches to determine whether *C. maritimum* deploys tolerance or resistance defense syndromes.

## 2. Materials and Methods

### 2.1. Study Area and Plant Material Sampling

**Figure 1:**
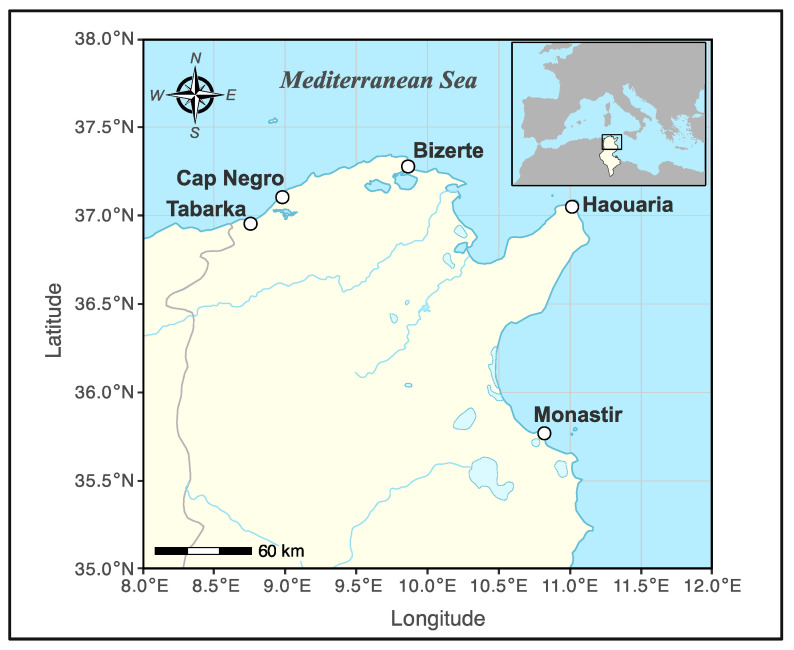
Geographic location of the five sampling sites of *Crithmum maritimum* L. along the Tunisian coast (Tabarka, Cap Negro, Bizerte, Haouaria, and Monastir).

The study was carried out along the Tunisian coast (Figure 1). An initial survey was conducted to identify suitable populations of *Crithmum maritimum* L. Five different sites (Table 1) were chosen for sampling based on two main criteria: first, a large enough plant population to allow sampling without causing ecological harm, and second, proximity to the laboratory to ensure quick transport and high quality of the plant material.

Twenty individuals of *C. maritimum* were randomly selected from each of the five sites. Before collection, measurements of the entire plant were recorded in the field. Plants were then harvested with minimal disturbance, collecting only the aboveground organs (stems, leaves, umbels, and seeds) required for subsequent analyses. Root tissues were not included in the analyses.

### 2.2. Morphological Trait Measurements

Morphometric analysis was conducted on each 100 individuals (20 per site) to assess phenotypic variation. All linear measurements (Table 2) were recorded in millimeters (mm), and surface areas in square millimeters (mm^2^). For traits measured on leaves, umbels, and umbellets, five replicates per individual were analyzed for statistical robustness, while 20 seeds per individual were measured. The average value of these replicates was used for each individual in subsequent analyses.

This method follows established standards for plant morphometrics, where leaf area is calculated from measurable traits like length and width and expressed in mm^2^ [25]. Leaf size definitions in functional trait databases also depend on projected leaf surface area (mm^2^) as the primary descriptor for comparative analysis [26]. Additionally, using standardized descriptors for lengths, diameters, and surface traits aligns with modern frameworks of quantitative plant morphology that integrate geometry and topology to capture trait variation [27].

### 2.3. Insect Attack Assessment

In addition to plant sampling, herbivory pressure was quantified at all sites by assessing visible damage restricted to structural and reproductive organs—stems, umbels, and seeds. Each individual was first examined in the field for clear signs of insect activity, such as boring holes or partially consumed seeds, which were visible to the naked eye. To confirm the biotic origin of the lesions and avoid confusion with abiotic injuries, all individuals were inspected under a ×10 stereomicroscope (Loupe binoculaire HAST-3EAW, Conatex SARL, Sarreguemines, France). Representative micro-images of the confirmed damage are provided in Supplementary Materials Figure S1. The herbivory rate was calculated as the percentage of plants showing confirmed insect damage relative to the total number of surveyed individuals per site.

This protocol follows established approaches used for reproductive and stem herbivory quantification, where visual scoring combined with microscopic validation provides accurate estimates of damage incidence and severity [28,29,30]. Sampling for morphological, biochemical, and ionomic analyses was performed during the period of highest herbivory pressure to ensure that trait variations reflected the peak of insect activity. Concurrently, molecular identification (DNA barcoding) of insect specimens retrieved from damaged umbels and seeds is being conducted to confirm their taxonomic identity and elucidate their life-cycle characteristics.

### 2.4. Sample Preparation for Biochemical and Mineral Analysis

After morphological measurements, plants were dried in a food dehydrator (Princess 112380, Princess Home Appliances, Breda, Netherlands) at 45 °C until their weight remained constant. Drying below 50 °C is generally considered optimal for preserving bioactive compounds and volatile oils, as higher temperatures accelerate the thermal degradation of phenolics, flavonoids, and essential oils [31]. Furthermore, our previous work demonstrated that 45 °C is the most suitable temperature for *C. maritimum*, essential oil yield, and preserving its phytochemical composition [32]. The dried samples were then separated into stems, umbels, flowers- used only for biochemical analysis-, and seeds, and ground into fine powders. For each site, 20 individual samples were pooled by organ. This pooling method is standard in phytochemistry and mineral nutrition research, as it minimizes intra-population variability while offering a representative profile at the site level [33].

### 2.5. Determination of Phenolic Content and In Vitro Antioxidant Activities

#### 2.5.1. Total Polyphenol Content (TPC) and Total Condensed Tannins (TCT)

TPC was measured using the method according to Kessler et al. (2002) [34]. Gallic acid (GA ≥ 98% purity; Sigma-Aldrich, St. Louis, MO, USA) calibrated the standard (concentrations, R^2^ = 0.9987). The absorbance of reduced phosphomolybdate/tungstate complex, by total phenolics, was monitored at 725 nm (FlexA-200 microplate reader, ALLSHENG Instrument Co., Ltd., Hangzhou, China).

Results were expressed as mg of GA equivalents per g of dry weight (mg·g^−1^ DW). TCT was measured using the vanillin (≥99% purity; Sigma-Aldrich, St. Louis, MO, USA) –HCl (37%; Merck, Darmstadt, Germany) method [35]. (±)-Catechin (DE (≥98% purity; Sigma-Aldrich, St. Louis, MO, USA) served as the standard (concentrations, R^2^ = 0.996). Results were expressed as mg CE·g^−1^ DW.

#### 2.5.2. DPPH and ABTS Radical Scavenging Activities

DPPH• free radical scavenging activity was determined according to the protocol used by Crawley (1997) [36] with some modifications. Briefly, a 20 µL aliquot of plant extract was combined with 200 µL of DPPH (Sigma-Aldrich, St. Louis, MO, USA) ethanolic solution (0.1 mM). The reaction mixture was incubated in the dark at room temperature (~22–25 °C) for 30 min, and the absorbance was then measured at 515 nm.

The ABTS•+ free radical assay followed Strauss et al. (1999) [37] with some modifications to adapt the method for 96-well microplates. ABTS•+ radical cations were produced by mixing 7 mM ABTS (Sigma-Aldrich, St. Louis, MO, USA) with 2.45 mM potassium persulfate (Merck, Darmstadt, Germany) and incubating the mixture in the dark for 16 h. Before use, the solution was diluted with deionized water to an absorbance of 0.7 at 734 nm. Plant extract solution (20 µL) was combined with 200 µL of the ABTS•+ solution, and the absorbance was measured at 734 nm after 6 min. Results were expressed as Trolox equivalents (mg TE·g^−1^ DW).

### 2.6. Mineral Composition

After acid digestion (HNO_3_, 65%; Merck, Darmstadt, Germany), macro- and microelement contents (Na, K, Ca, Mg, P, and selected trace elements) were analyzed. Analyses were conducted using ICP–AES (Horiba Scientific Activa, Kyoto, Japan) according to the manufacturer’s guidelines [38], and ICP–MS (Analytik, Jena, Germany) for trace element quantification, using validated protocols [39].

### 2.7. Lipophilic Fraction Extraction and GC–MS Analysis

The lipophilic fraction was extracted from dried seeds and umbels of *C. maritimum* using n-hexane in a Soxhlet apparatus (behrotest® Soxhlet Extraction, 8-positions; behr Labor-Technik GmbH, Düsseldorf, Germany) following classical total lipids determination protocols [40,41].

Extracts were concentrated at 40 °C under reduced pressure and stored at −20 °C until analysis. Fatty acids were converted to methyl esters (FAMEs) using 3% sodium methoxide (NaOCH3) and sulphuric acid [42]. GC–MS analysis was performed on a capillary HP-88 column (60 m × 0.25 mm × 0.20 µm Agilent Technologies, Santa Clara, USA) with helium as carrier gas.

Oven program: 120 °C (1 min), 10 °C min^−1^ to 175 °C, then 3 °C min^−1^ to 220 °C (10 min hold). Injector and detector temperatures: 250 °C. The MS was operated in EI mode (70 eV), scanning *m*/*z* 50–550. Compounds were identified by comparison with NIST library spectra and retention indices calculated with a homologous n-alkane series [43,44,45]. Fatty acid composition was expressed as a percentage of total FAMEs (area normalization).

### 2.8. Statistical Analysis

All statistical analyses were carried out in Python v3.11.9 (Python Software Foundation, Wilmington, DE, USA). The following libraries were used: pandas and numpy for data management and numerical operations; scipy.stats and statsmodels for univariate statistics, including one-way ANOVA and Tukey’s HSD post hoc tests; scikit-learn for multivariate analyses (PCA and PLS-DA); pingouin for correlation analyses; and seaborn and matplotlib for data visualization.

Prior to analysis, all variables were examined for normality and homogeneity of variances using the Shapiro–Wilk and Levene’s tests, respectively. Data that did not meet ANOVA assumptions were log_10−_ or square-root-transformed to stabilize variance and approximate normal distribution. ANOVA outputs were interpreted on the transformed scale, while mean values are presented on the original scale (mean ± SD) for clarity.

Differences in herbivory rates among the five sites (*n* = 100 individuals in total, 20 per site) were assessed using one-way ANOVA, followed by Tukey’s HSD test to identify statistically homogeneous groups. Based on these results, sites were classified into Low-Herbivory (Tabarka, Bizerte) and High-Herbivory (Cap Negro, Haouaria, Monastir, Tunisia) categories.

Spearman’s rank correlations were used to explore relationships between morphological traits and herbivory intensity. Principal Component Analysis (PCA) was used for exploratory ordination of the lipophilic trait dataset. Partial Least Squares Discriminant Analysis (PLS-DA) was then applied to identify discriminant variables separating Low- and High-Herbivory groups, with Variable Importance in Projection (VIP > 1) values indicating the most influential traits.

Complementary analyses included hierarchical clustering (Ward’s method, Euclidean distance) and clustered heatmaps to visualize trait associations. A distance-based redundancy analysis (dbRDA) integrated morphological, biochemical, mineral, and lipophilic datasets with climatic variables (precipitation and bioclimatic tier) to partition the variance explained by herbivory, climate, and their interaction.

All quantitative variables were centered and standardized prior to PCA, PLS-DA, clustering, and dbRDA. Model reproducibility was ensured by setting random_state = 42. The reliability of the PLS-DA model was further validated through 10-fold cross-validation and permutation testing (n = 100) to confirm that discrimination between herbivory groups was not driven by random chance.

## 3. Results

### 3.1. Herbivory Pressure Varies Significantly Across Sites

**Figure 2:**
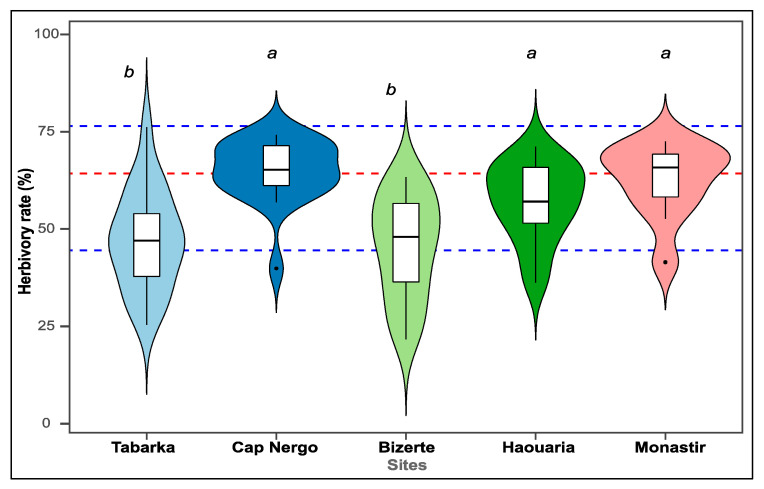
Herbivory pressure on *C. maritimum* populations. Mean percentage of herbivore damage across the five study sites. Sites are separated into Low- (Tabarka, Bizerte) and High-Herbivory (Haouaria, Monastir, Cap Negro) groups. Dashed lines represent the overall median (red) and first and third quartiles (blue). The width of each violin plot reflects the density of occurrence data points, and the boxes indicate the interquartile range. Different letters indicate statistically significant differences among sites (*p* ≤ 0.05).

The intensity of insect herbivory varied substantially among the five sampled populations, ranging from 51.08% in Tabarka to 73.23% in Cap Negro. One-way ANOVA revealed highly significant differences in herbivory rates among sites (*F*_4,95_ = 13.4; *p* < 0.001). Tukey’s HSD post hoc test indicated that Tabarka and Bizerte formed a homogeneous subset (*p* > 0.05), clearly distinct from the other three populations (Haouaria, Monastir, and Cap Negro; all *p* < 0.05). Accordingly, sites were grouped into Low-Herbivory (Tabarka, Bizerte) and High-Herbivory (Haouaria, Monastir, Cap Negro) categories (Figure 2, Table 3).

Given this clear separation into Low- and High-Herbivory groups, we hypothesized that *C. maritimum* may adopt distinct adaptive strategies in response to contrasting levels of insect pressure. To test this hypothesis, integrative multivariate analyses were performed to identify the traits most strongly associated with herbivory attack.

### 3.2. Effect of Herbivory on Morphological Traits

**Figure 3:**
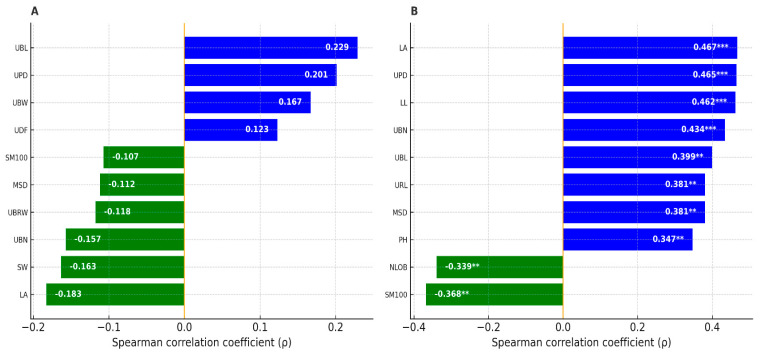
Spearman’s correlations coefficients (ρ) between the number of attacked fruits per plant and morphological traits in low-herbivory sites (**A**, n = 40) and high-herbivory sites (**B**, n = 60). Bars are blue for positive and green for negative correlations; values inside bars are ρ. Asterisks denote significance: ** = *p* < 0.01; *** = *p* < 0.001.

Using the individual-level data, we assessed the association between herbivory intensity—expressed as the number of attacked fruits per plant—and each morphological trait with Spearman’s rank correlations. Analyses were performed separately for low-herbivory sites (Tabarka, Bizerte; *n* = 40) and high-herbivory sites (Cap Negro, Haouaria, Monastir; *n* = 60) (Figure 3).

In low-herbivory sites, no correlation reached significance; the most significant effects were minor, with weak positive tendencies for umbel bract length (UBL; ρ = 0.229, *p* = 0.156) and peduncle diameter (UPD; ρ = 0.201, *p* = 0.213), and weak negative tendencies for leaf area (LA; ρ = −0.183, *p* = 0.259) and seed width (SW; ρ ≈ −0.163, *p* > 0.30). In high-herbivory sites, several traits correlated significantly with herbivory. The number of attacked fruits increased with leaf size and more robust inflorescence structures, including LA (ρ = 0.467, *p* = 1.7 × 10^−4^ ***), UPD (ρ = 0.465, *p* = 1.8 × 10^−4^ ***), leaf length LL (ρ = 0.462, *p* = 2.0 × 10^−4^ ***), the number of umbel bracts UBN (ρ = 0.434, *p* = 5.3 × 10^−4^ ***), as well as UBL (ρ = 0.399, *p* = 0.0016), ray length URL (ρ = 0.381, *p* = 0.0027), main stem diameter MSD (ρ = 0.381, *p* = 0.0027), plant height PH (ρ = 0.347, *p* = 0.0066), leaf width LW (ρ = 0.339, *p* = 0.0081) and the number of umbellet bracteoles UNBR (ρ = 0.333, *p* = 0.0093). In this context, two traits were negatively associated with herbivory: 100-seed weight SM100 (ρ = −0.368, *p* = 0.0039) and the number of leaf lobes NLOB (ρ = −0.339, *p* = 0.0081). Overall, the absence of detectable associations under low herbivory and the emergence of multiple moderate correlations under high herbivory indicate a context-dependent coupling between plant architecture and herbivore damage.

### 3.3. Effect of Herbivory on Biochemical Traits

**Figure 4:**
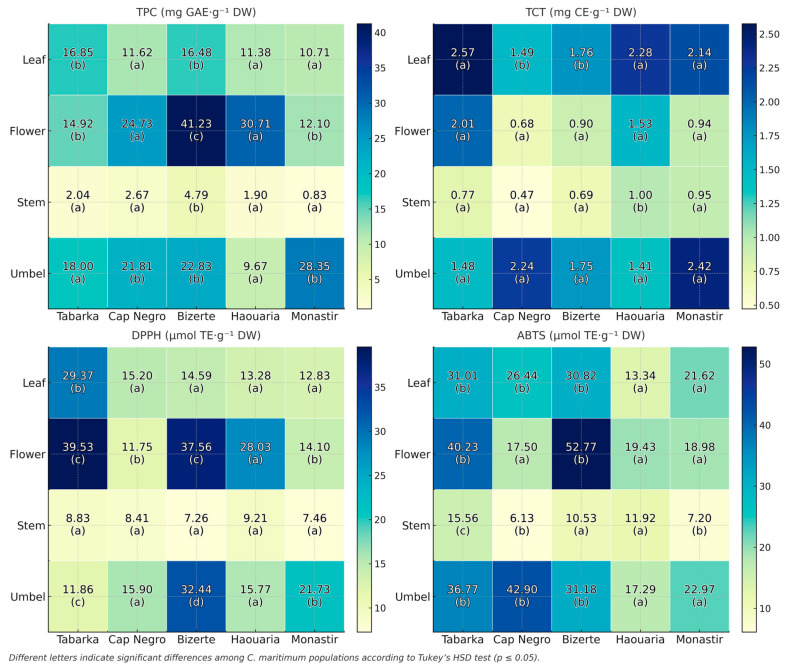
Effect of herbivory on biochemical traits in low- and high-herbivory populations. Heatmaps show site-level means for TPC (mg GAE·g^−1^ DW), TCT (mg CE·g^−1^ DW), DPPH (µmol TE·g^−1^ DW), and ABTS (µmol TE·g^−1^ DW) across four organs (Leaf, Flower, Stem, Umbel). Different letters indicate significant differences among sites according to Tukey’s HSD test (*p* ≤ 0.05).

Biochemical traits were quantified in four organs (Leaf, Flower, Stem, Umbell) across Low (Tabarka, Bizerte) and High herbivory sites (Cap Negro, Haouaria, Monastir). Values are expressed as mean ± SD (n = 4) and are summarized in Figure 4.

In the Low herbivory group, leaves exhibited TPC values ranging from 14.08 ± 2.72 to 19.92 ± 2.00 mg GAE·g^−1^ DW, while ABTS activities varied between 23.99 ± 3.00 and 39.72 ± 2.10 µmol TE·g^−1^ DW. Flowers displayed higher TPC, reaching 44.67 ± 0.77 mg GAE·g^−1^ DW in Bizerte, while umbels showed moderate levels, ranging from 14.92 ± 1.80 to 25.25 ± 2.10 mg GAE·g^−1^ DW. Stems had the lowest values overall, with TPC between 1.25 ± 0.52 and 5.75 ± 0.83 mg GAE·g^−1^ DW.

In the High herbivory group, leaves had lower phenolic contents, ranging from 9.17 ± 2.27 to 13.50 ± 1.21 mg GAE·g^−1^ DW, with ABTS values between 11.49 ± 1.90 and 28.66 ± 2.30 µmol TE·g^−1^ DW. Flowers were highly variable, from 12.00 ± 0.17 mg GAE·g^−1^ DW in Monastir to 40.50 ± 1.53 mg GAE·g^−1^ DW in Haouaria. Umbels showed the broadest range of values, from 8.42 ± 1.54 mg GAE·g^−1^ DW in Haouaria to 36.25 ± 3.73 mg GAE·g^−1^ DW in Monastir. Stem extracts consistently displayed the lowest activities, with TPC < 4.0 mg GAE·g^−1^ DW and antioxidant capacities (DPPH, ABTS) < 16 µmol TE·g^−1^ DW.

The heatmap shows distinct differences in biochemical profiles between Low and High herbivory sites. In the Low group (Tabarka, Bizerte), flowers and umbels generally had higher phenolic and antioxidant levels than stems, which consistently recorded the lowest values across all traits.

In the High herbivory group (Cap Negro, Haouaria, Monastir), significant site-dependent differences were observed. Haouaria flowers showed the highest TPC values, while Monastir umbels recorded the highest phenolic contents and antioxidant activities (TPC, ABTS). Conversely, Haouaria umbels displayed the lowest phenolic and antioxidant levels among the High sites. Stems remained the least active organs across all sites.

The heatmap highlights that reproductive organs (flowers and umbels) are the most variable across sites, while stems consistently show low biochemical values regardless of herbivory levels.

Building on these biochemical patterns, we next examined the mineral composition of *C. maritimum* organs across the five sites to determine whether macro- and micro-element profiles also differed between populations exposed to low versus high herbivory pressure.

### 3.4. Effect of Herbivory on Mineral Composition

The mineral composition analysis revealed apparent differences among *C. maritimum* organs and between populations subjected to low versus high herbivory pressure (Table 4). Potassium was the most abundant element in low-herbivory sites (Tabarka, Bizerte), reaching 890.6 mg·g^−1^ DW in umbels from Tabarka. Calcium and magnesium were also consistently high in leaves and stems, with Tabarka leaves containing up to 515.2 mg·g^−1^ DW Ca and stems reaching 515.2 mg·g^−1^ DW Mg.

On the other hand, populations exposed to high herbivory (Cap Negro, Haouaria, Monastir) showed increased levels of phosphorus and sodium, especially in reproductive organs. For example, umbels from Monastir had 65.8 mg·g^−1^ DW P and 623.1 mg·g^−1^ DW Na, significantly higher than those found in populations with low herbivory.

Trace elements involved in plant defense, such as iron, zinc, manganese, and copper, also varied depending on the site. Iron concentrations were highest in flowers from Cap Negro (9.8 mg·g^−1^ DW), while zinc and manganese were more abundant in Tabarka leaves (0.46 mg·g^−1^ DW Zn and 0.21 mg·g^−1^ DW Mn).

Following the mineral composition analysis, we investigated the lipophilic fraction (GC–MS) to evaluate whether herbivory pressure modulates fatty acids, sterols, terpenes, and phenylpropanoids.

### 3.5. Effect of Herbivory on Lipophilic Fraction (GC–MS)

**Figure 5:**
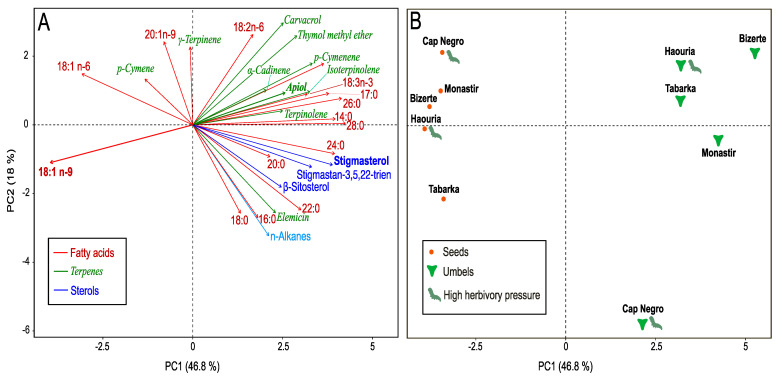
PCA of the lipophilic fraction of *C. maritimum* under contrasting herbivory pressure. (**A**) Loadings plot highlighting the main discriminant compounds. (**B**) Scores plot showing population separation by herbivory level and site. PCA performed on 15 samples (five sites × three replicates). PC1 and PC2 explain 46.8% and 18.0% of variance, respectively. Statistical validation by ANOVA (Tukey HSD, *p* ≤ 0.05) confirmed significant site-level differences.

The GC–MS profiling revealed site- and organ-specific signatures further structured by herbivory level. The loadings plot (Figure 5A) highlighted the main compounds contributing to the observed differentiation among sites. Separation along PC1 was mainly associated with sterols (β-sitosterol, stigmasterol, stigmastan-3,5,22-trien) and terpenes (γ-terpinene, terpinolene, p-cymene/p-cymenene, isoterpinolene), whereas PC2 was driven by saturated fatty acids (C14:0, C16:0, C17:0, C18:0) and apiol.

The scores plot (Figure 5B) displayed a clear separation between low-herbivory sites (Tabarka, Bizerte) and high-herbivory populations (Cap Negro, Haouaria, Monastir), with seeds and umbels forming distinguishable clusters within each group. The first two principal components explained 46.8% (PC1) and 18.0% (PC2) of the total variance.

To statistically validate the patterns observed in the PCA, we compared the relative abundances of the main lipophilic classes (SFA, MUFA, PUFA, sterols, terpenes, and apiol) among sites using ANOVA followed by Tukey’s post hoc test. The loadings plot (Figure 5A) identified the compounds most responsible for the observed differentiation, while the scores plot (Figure 5B) confirmed the separation of populations according to herbivory level and site.

ANOVA did not detect statistically significant differences among sites for apiol (*p* = 0.35). Apiol levels were 17.42 ± 0.45% in Monastir, 5.85 ± 2.21% in Cap Negro, 6.78 ± 8.04% in Haouaria, 9.44 ± 8.10% in Tabarka, and 7.99 ± 4.05% in Bizerte (Table 5).

Sterols were approximately ~1.50% in Bizerte and Tabarka and ~1.30% in Monastir, Haouaria, and Cap Negro. Terpenes ranged from 0.14 ± 0.12% in Cap Negro to 0.79 ± 1.05% in Monastir.

When populations were grouped by herbivory level, apiol averaged 10.7% in high-herbivory sites and 8.7% in low-herbivory sites. Sterols averaged 1.91% in high-herbivory sites and 2.55% in low-herbivory sites. PUFA averaged 0.08% in high-herbivory sites and 0.14% in low-herbivory sites. Terpenes averaged 1.32% in high-herbivory sites and 1.11% in low-herbivory sites.

Together, these results describe the variation in apiol, sterols, PUFA, and terpenes across herbivory levels, as reflected in the PCA and summarized in Table 5.

To further verify the difference between low- and high-herbivory populations, we used Partial Least Squares Discriminant Analysis (PLS-DA), a supervised technique often employed in metabolomics. This method identifies the compounds that most influence the separation between groups using Variable Importance in Projection (VIP) scores.

**Figure 6:**
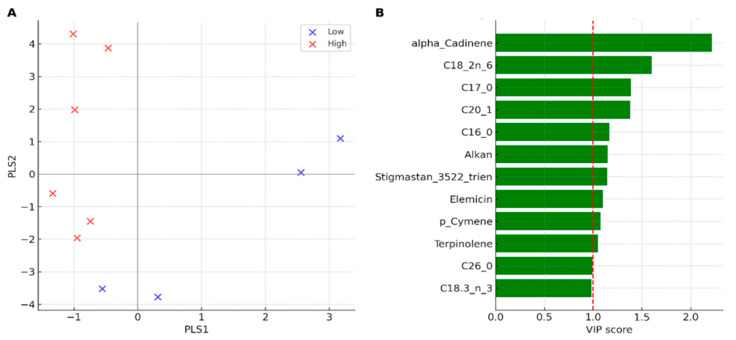
PLS-DA of the lipophilic fraction of *C. maritimum* under contrasting herbivory pressure. (**A**) PLS-DA scores plot showing clear discrimination between low- (Tabarka, Bizerte) and high-herbivory (Cap Negro, Haouaria, Monastir) populations. (**B**) VIP scores (top 12 compounds) identifying the main discriminants, with α-cadinene, linoleic acid (C18:2 n-6), C17:0, C20:1, and palmitic acid (C16:0) emerging as the most influential variables (VIP > 1).

The PLS-DA clearly separated low-herbivory (Tabarka, Bizerte) and high-herbivory populations (Cap Negro, Haouaria, Monastir) along the first two components (Figure 6A), confirming the grouping previously observed in the PCA (Figure 5A). The supervised model highlighted the strong contribution of specific compounds to discrimination. VIP scores identified α-cadinene (VIP ≈ 2.20), linoleic acid C18:2 n-6 (VIP ≈ 1.58), heptadecanoic acid C17:0 (VIP ≈ 1.37) and C20:1 (VIP ≈ 1.36) as the main variables distinguishing the groups, followed by palmitic acid C16:0 (VIP ≈ 1.15) and stigmastan-3,5,22-trien (VIP ≈ 1.13) (Figure 6B). Apiol (VIP ≈ 0.80) showed a lower contribution to group separation in this model. This supervised analysis is consistent with the patterns observed in the PCA and summarized in Table 5.

To further visualize how discriminant lipophilic compounds group populations according to herbivory level, a hierarchical clustering analysis (HCA) combined with a heatmap was performed using the top variables identified by PLS-DA (VIP > 1.0).

**Figure 7:**
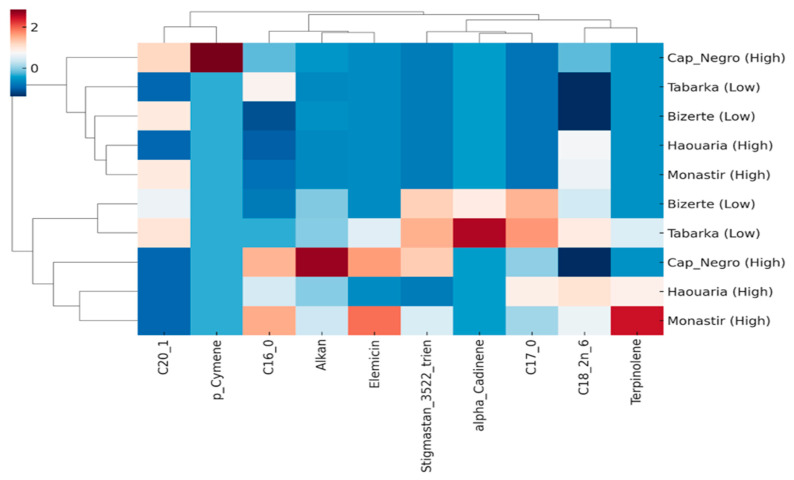
Hierarchical clustering analysis (HCA) and heatmap of discriminant lipophilic compounds (VIP > 1). Rows represent sites (low-herbivory: Tabarka, Bizerte; high-herbivory: Cap Negro, Haouaria, Monastir), and columns represent the most discriminant compounds identified by PLS-DA. Clustering clearly separates low- and high-herbivory populations, with Monastir showing a distinct lipophilic signature within the high-herbivory group.

The heatmap and hierarchical clustering clearly distinguished low-herbivory sites (Tabarka, Bizerte) from high-herbivory populations (Cap Negro, Haouaria, Monastir) based on the abundance of discriminant compounds (Figure 7). The clustering dendrogram grouped the two low-herbivory sites, while Monastir was separated within the high-herbivory group, reflecting its particularly high apiol and terpene contents.

At the compound level, α-cadinene and linoleic acid (C18:2 n-6) were strongly linked to high-herbivory areas, while sterols (stigmastan-3,5,22-trien) and palmitic acid (C16:0) were more common in low-herbivory populations. Elemicin, p-cymene, and terpinolene showed intermediate levels, contributing to subtle differences among high-herbivory sites.

This clustering pattern supports the results of PCA, ANOVA, and PLS-DA, confirming that lipophilic traits are consistently shaped by herbivory pressure and that apiol and specific terpenes are key indicators of high herbivory.

Given the transparent climatic gradient among the study sites, we incorporated bioclimatic factors—specifically, mean annual precipitation and bioclimatic tier—into a multivariate analysis that combines morphological, biochemical, mineral, and lipophilic traits.

### 3.6. Integrative Analysis of Herbivory-Related Traits and Bioclimatic Context

**Figure 8:**
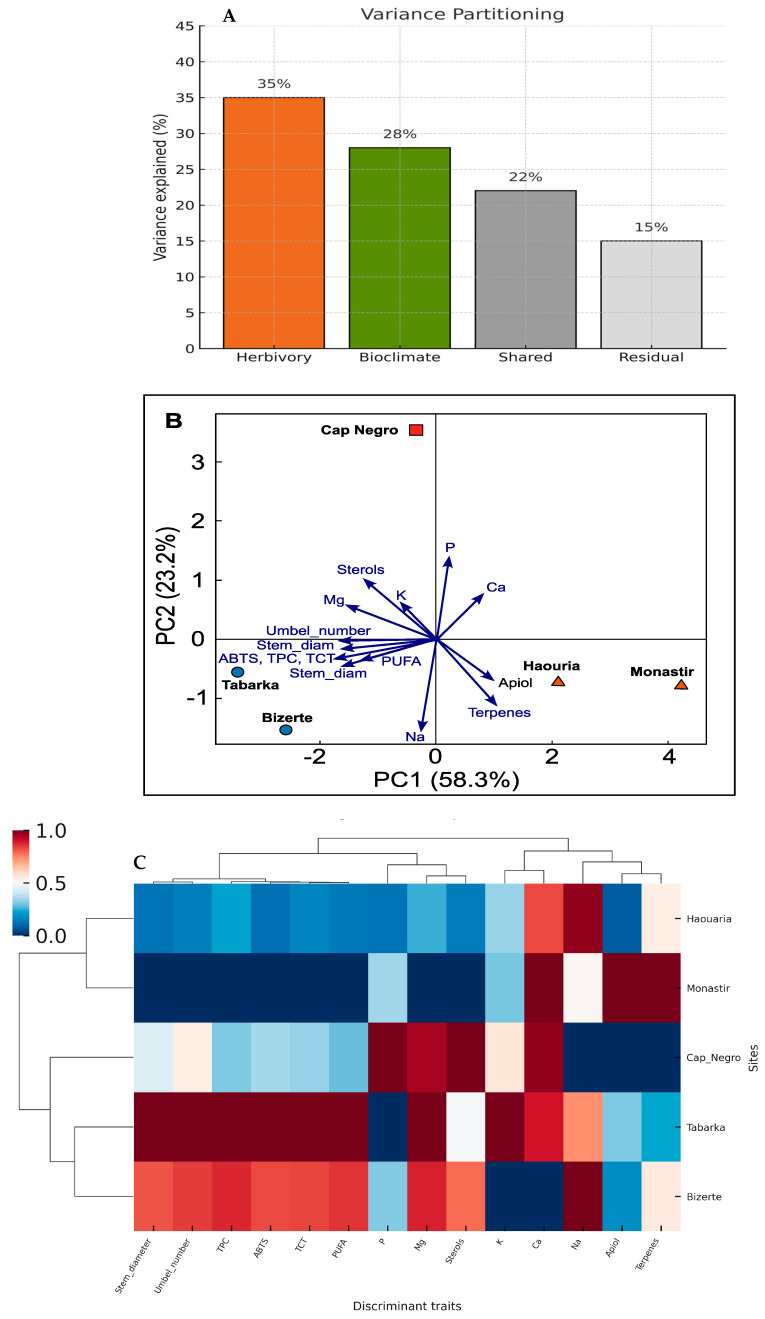
Integrative multivariate analysis of herbivory-related traits and bioclimatic context. (**A**) Variance partitioning showing the unique and shared contributions of herbivory pressure and bioclimatic tier to the variance in trait composition. (**B**) dbRDA ordination of site means, with points colored by herbivory level (Low vs. High) and shaped by bioclimatic tier (Sub-humid, Transitional, Semi-arid). Trait vectors indicate the main discriminant variables. (**C**) Heatmap and hierarchical clustering of z-scored discriminant traits (morphological, biochemical, mineral, and lipophilic), showing consistent grouping of sub-humid low-herbivory versus semi-arid high-herbivory populations, with Cap Negro in an intermediate position.

The integrative analysis combining morphological, biochemical, mineral, and lipophilic traits with bioclimatic parameters offered a comprehensive view of the factors driving trait variability across *C. maritimum* populations (Figure 8).

Variance partitioning (Figure 8A) showed that herbivory pressure alone explained 35% of the observed variance, while the bioclimatic tier (sub-humid, transitional, semi-arid) accounted for 28%. Their shared contribution comprised 22%, and the remaining variance was limited to 15%. These results demonstrate that both herbivory and climate are strong, partly overlapping factors shaping the defense syndromes of *C. maritimum*.

The dbRDA ordination (Figure 8B) further highlighted a clear separation of sites based on herbivory and climate. Populations from low-herbivory, sub-humid sites (Tabarka and Bizerte) clustered together and were characterized by higher levels of structural traits (stem diameter, number of umbels), biochemical antioxidants (TPC, TCT, ABTS), and mineral nutrients (Ca, Mg, K), as well as sterols and PUFA. In contrast, high-herbivory, semi-arid sites (Haouaria and Monastir) grouped and were strongly associated with increased levels of Apiol and terpenes, along with higher Na and P levels, which contrasted with their lower Ca and Mg contents. These opposing trait patterns reflect two strategies: a tolerance strategy in sub-humid, low-herbivory populations versus a resistance strategy in semi-arid, high-herbivory populations.

The heatmap and hierarchical clustering (Figure 8C) supported these patterns by consistently distinguishing low-herbivory sub-humid populations (Tabarka, Bizerte) from high-herbivory semi-arid populations (Haouaria, Monastir). Interestingly, despite being categorized in this group, Cap Negro did not cluster tightly with the high-herbivory group. Instead, it occupied an intermediate position, reflecting its transitional bioclimatic conditions and mixed trait profile.

Specifically, Cap Negro showed moderate levels of Ca and Mg, intermediate Apiol and terpenes, and increased but not extreme Na and P, placing it between the two defense syndromes. This intermediate position emphasizes the herbivory responses’ gradual and flexible nature, showing that *C. maritimum* does not follow a strict Low/High dichotomy but demonstrates adaptive flexibility along climatic and herbivory gradients.

Together, these integrative results reinforce the findings from previous Section 3.2, Section 3.3, Section 3.4 and Section 3.5 and show that *C. maritimum* populations use two distinct but complementary defense strategies, influenced by both herbivory intensity and bioclimatic conditions. This offers a solid framework for understanding herbivory-related trait syndromes’ ecological and adaptive importance in coastal halophytes.

These integrative patterns form the cornerstone for interpreting how *C. maritimum* balances tolerance and resistance strategies under varying ecological pressures. The following discussion will contextualize these findings within the broader framework of plant defense theory and halophyte adaptation in Mediterranean ecosystems.

## 4. Discussion

The integration of morphological, biochemical, mineral, and lipophilic traits observed in *Crithmum maritimum* L. reveals two complementary strategies of defense against herbivory, modulated by the bioclimatic gradient. Mechanistically, resistance involves processes that reduce damage—including the accumulation of deterrent metabolites, lignified tissues, and the activation of hormone-mediated signaling pathways such as jasmonic acid (JA), salicylic acid (SA), and ethylene—whereas tolerance refers to the ability to recover performance after damage through increased photosynthetic compensation, regrowth, or enhanced reproductive effort [46,47,48]. These mechanisms represent distinct yet interacting dimensions of plant adaptation to herbivory, each associated with specific metabolic costs and ecological advantages [49,50,51].

Herbivory rates significantly differed among sites (F_4.95_ = 13.4, *p* < 0.001), ranging from 51% at Tabarka to 73% at Cap Negro. The clustering analysis distinguished two groups: low-herbivory populations (Tabarka, Bizerte) and high-herbivory populations (Cap Negro, Haouaria, Monastir).

Populations exposed to milder herbivory, located in sub-humid coastal environments, exhibited a tolerance-oriented syndrome. Morphologically, these plants showed thicker stems, larger peduncles, and greater structural cohesion—traits that enhance mechanical resistance to partial tissue loss and maintain canopy integrity [52,53]. Such architecture facilitates hydraulic stability and sustained carbon assimilation, supporting tolerance through continuous resource acquisition [54].

Biochemically, low-herbivory populations maintained high antioxidant capacity, with elevated total phenolics (TPC), tannins (TCT), and radical-scavenging activities (ABTS, DPPH). This antioxidant machinery minimizes oxidative damage caused by wounding and stabilizes chloroplast membranes [55,56]. Phenolic compounds also serve as ROS buffers and signaling intermediates, linking oxidative stress to defense gene activation [57].

Ionomic profiles revealed enrichment in Ca, Mg, and K, elements associated with cell-wall rigidity, stomatal regulation, and redox co-factor balance [58,59]. Calcium plays a central role as a secondary messenger in wounding and JA induction [60], while Mg and K optimize photosynthetic recovery and osmotic control, enhancing tolerance through physiological compensation [61]. These integrated traits define a proactive defense state, in which energy is invested in structural stability and oxidative homeostasis rather than inducible metabolite production. This aligns with theoretical predictions that plants in predictable, low-damage environments favor maintenance-based tolerance over costly resistance [62].

In contrast, populations subjected to higher herbivory in semi-arid sites expressed a resistance-dominated syndrome. Morphological correlations indicated that the number of attacked fruits was positively associated with stem diameter (MSD), peduncle diameter (UPD), and umbel traits (URL, UBN, UBL), reflecting reproductive over-investment under stress [63]. This pattern, often termed “compensatory reproduction”, represents an adaptive shift toward fitness assurance when vegetative tissues are recurrently damaged [64,65].

At the biochemical level, these populations accumulated lipophilic and terpenoid compounds, including α-cadinene, linoleic acid (C18:2 n-6), eicosenoic acid (C20:1), and terpinolene—molecules known to function as direct toxins, feeding deterrents, or precursors of HIPVs (herbivore-induced plant volatiles) [66,67,68]. The activation of oxylipin pathways and the interplay between JA and oxylipin signaling enhance rapid resistance induction [69]. Increased unsaturated fatty acids, particularly PUFAs, may further contribute to membrane fluidity and stress signal propagation [70].

These metabolic investments are coupled with an ionomic shift toward Na and P accumulation, characteristic of xerohalophytic adaptation. Phosphorus supports ATP-dependent biosynthetic processes and the regeneration of NADPH for redox balance, whereas Na contributes to osmotic maintenance under arid stress [71,72]. Such traits indicate an energetic reorganization favoring secondary metabolism and volatile emission at the expense of growth. High-herbivory populations therefore represent a reactive defense state, where chemical deterrence is prioritized once morphological tolerance limits are exceeded [73,74].

Cap Negro populations, situated at the climatic and ecological transition between the two groups, exhibited a hybrid phenotype. They combined morphological robustness (thick stems and peduncles) with moderate antioxidant capacity and the co-accumulation of both sterols (β-sitosterol, stigmastan-3,5,22-trien) and terpenoids [75]. This intermediate configuration suggests context-dependent plasticity, where defense allocation is fine-tuned according to fluctuating herbivory and microclimatic stress [76,77]. The coexistence of tolerance and resistance traits at Cap Negro reflects a dynamic optimization strategy rather than a fixed defense type—an outcome consistent with evolutionary models of adaptive integration [78].

Variance partitioning revealed that herbivory accounted for 35% of total trait variance, climate for 28%, and their shared contribution for 22% (R^2^adj = 0.74, *p* < 0.001), underscoring the synergistic regulation of defense expression by biotic and abiotic factors [79,80]. This interaction illustrates how environmental context not only constrains but also facilitates defense evolution by modulating resource availability, oxidative signaling, and ionomic balance [81,82].

At the evolutionary level, *C. maritimum* demonstrates stabilizing selection favoring flexible genotypes capable of shifting between tolerance and resistance. This adaptability is likely mediated by epigenetic mechanisms—including DNA methylation and histone modifications—that enable rapid transcriptional reprogramming of stress-related genes [83,84]. The involvement of WRKY and bZIP transcription factors further supports coordinated control between growth, metabolism, and defense allocation [85].

In conclusion, *Crithmum maritimum* L. exemplifies an evolutionarily resilient halophyte capable of integrating morphological, biochemical, and metabolic responses across contrasting environments. Morphological and antioxidant tolerance predominates under low herbivory, while chemical resistance and metabolic activation prevail under high herbivory. The Cap Negro population bridges these syndromes through flexible, plastic integration. Together, these findings demonstrate that herbivory, modulated by climate, acts as a selective force shaping a mosaic of adaptive defense strategies, ensuring the persistence of *C. maritimum* in Mediterranean coastal ecosystems. Although the present analyses demonstrate statistically significant associations between herbivory incidence and multiple biochemical and mineral traits, these relationships remain observational. Future experimental approaches would be required to verify direct causal links under controlled conditions.

## 5. Conclusions

Overall, this study shows that *Crithmum maritimum* L. employs two distinct and opposing defense strategies against insect herbivory: a biochemical resistance approach driven by Apiol, Dillapiole, phenolics, and tannins, and a tolerance strategy that emphasizes mineral allocation, seed enrichment, and morphological compensation. These findings highlight C. *maritimum* as an excellent model for studying the evolutionary ecology of plant defense and provide valuable insights for its conservation and domestication.

Importantly, this work presents the first documentation of insect herbivory on *C. maritimum*, a halophytic species of growing ecological and economic significance. Consequently, it may serve as an early warning for Mediterranean countries, where this species is traditionally harvested and increasingly valued, suggesting that herbivory pressure may pose an emerging threat to its wild populations and potential cultivation.

By documenting this phenomenon and revealing the duality of defense syndromes, this article can be regarded as a precursor to future research on the eco-evolutionary dynamics of plant–insect interactions in halophytes. Further studies, including identifying herbivore species and integrating genomic and metabolomic approaches, will deepen our understanding and guide strategies for conservation, domestication, and sustainable use of *C. maritimum*.

Finally, identifying the herbivorous insect will offer practical insights for conservation and food security planning in Mediterranean coastal areas.

## Figures and Tables

**Table 1:** Geographic coordinates and climatic characteristics of *Crithmum maritimum* L. sampling sites along the Tunisian coastline.

Site	Latitude (° N)	Longitude (° E)	Altitude (m, a.s.l.)	Annual Temperature (°C)	Annual Precipitation (mm)
Tabarka	36.954	8.758	5	18.8	1010.4
Cap Negro	37.104	8.982	0	18.6	948
Bizerte	37.278	9.864	5	19	571
Haouaria	37.05	11.014	21	19.7	481.9
Monastir	35.769	10.819	10	20.6	383.7

“Climatic data are averages from INM/INS (2015–2023) for Bizerte, Haouaria, Monastir, and Tabarka. Cap Negro values are proxies from Tabarka, reported by UNEP/MAP (long-term INM data)”.

**Table 2:** Description of the morphological trait groups evaluated in *C. maritimum*.

Trait Group	Recorded Traits (Abbreviations and Units)
Whole-Plant Traits	Height (PH, mm), length (PL, mm), and width (PW, mm).
Stem Traits	Main stem diameter (MSD, mm).
Leaf Traits	Length (LL, mm), width (LW, mm), number of lobes (NLOB), projected leaf area (LA, mm^2^), Montgomery shape factor (ks) *, average lobe area (ALOB) **
Umbel Traits	Peduncle length (UPL, mm), peduncle diameter (UPD, mm), number of rays (URN), length of rays (URL, mm), thickness of the rays (URT, mm), number of bracts forming the involucre (UBN), length of bracts (UBL, mm), width of individual bracts (UBW, mm).
Umbellet Traits	Number of pedicels for the secondary clusters (UNPED), number of bracteoles forming the involucel (UNBR), length of bracteole (UBRL, mm), width of bracteole (UBRW, mm)
Seed Traits	Seed length (SL, mm), seeds width (SW, mm), surface area (mm^2^), perimeter (SPER, mm), weight of 100 seeds (SM100, g).

* ks is calculated using the formula LA / (LL × LW); ** ALOB = LA/ NLOB [24].

**Table 3:** Herbivory rates across sites and classification into herbivory groups.

Site	Herbivory Rate (%) *	Herbivory Group
Tabarka	51.08	Low
Bizerte	54.92	Low
Haouaria	68.81	High
Monastir	70.64	High
Cap Negro	73.23	High

Mean herbivory rate (%) and corresponding group classification. * Herbivory rates per site are derived from * B.H.’s ongoing Master’s dataset (INAT).

**Table 4:** (**a**) Mineral composition (mg·g^−1^ DW) of *C. maritimum* organs from low-herbivory sites (Tabarka and Bizerte). (**b**) Mineral composition (mg·g^−1^ DW) of *C. maritimum* organs from high-herbivory sites (Cap Negro, Haouaria, and Monastir).

(a)										
**Site**	**Organ**	**K**	**Ca**	**Mg**	**P**	**Na**	**Fe**	**Zn**	**Mn**	**Cu**
Tabarka	Flower	619.74	509.29	97.11	15.08	616.61	4.68	0.42	0.23	0.09
	Leaf	577.59	515.25	515.25	19.2	513.9	4.13	0.46	0.21	0.06
	Stem	784.99	227.32	68.55	21.72	579.6	2.2	0.48	0.07	0.08
	Umbell	890.63	392.22	99.83	41.08	623.06	3.21	0.46	0.17	0.13
Bizerte	Flower	404.15	271.27	109.13	35.8	194.32	3.9	0.46	0.31	0.11
	Leaf	503.45	413	413	14.66	857.63	8.79	0.46	0.36	0.06
	Stem	746.75	221.77	76.71	33.01	682.84	2.33	0.29	0.2	0.1
	Umbell	727.05	263.67	118.53	38.37	669.65	4.22	0.44	0.4	0.14
(**b**)										
**Site**	**Organ**	**K**	**Ca**	**Mg**	**P**	**Na**	**Fe**	**Zn**	**Mn**	**Cu**
Cap Negro	Flower	872.77	433.85	52.35	52.75	444.41	3.97	0.73	0.32	0.19
	Leaf	887.93	542.37	542.37	64.69	541.61	9.84	0.62	0.42	0.15
	Stem	773.94	270.63	24.78	19.55	780.54	1.45	0.31	0.1	0.08
	Umbell	514.09	411.52	133.24	39.45	372.65	9.58	0.51	0.42	0.16
Haouaria	Flower	686.87	340.86	76.39	34.86	468.28	7.51	0.49	0.6	0.16
	Leaf	607.9	574.42	133.02	28.5	937.28	17.76	0.54	0.91	0.17
	Stem	710.83	314.19	83.34	16.65	557.72	9.66	0.41	0.35	0.13
	Umbell	774.38	357.52	94.52	27.54	433.41	19.74	0.5	0.39	0.19
Monastir	Flower	492.99	646.17	68.65	32.47	446.46	7.38	0.29	0.29	0.06
	Leaf	835.06	335.05	74.99	28.58	941.51	3.66	0.5	0.22	0.11
	Stem	716.86	291.35	45.2	32.46	344.67	1.32	0.33	0.08	0.1
	Umbell	695.84	399.72	65.01	31.11	542.27	3.48	0.59	0.23	0.17

**Table 5:** Relative abundance (mean ± SD, %) of selected lipophilic classes in *C. maritimum* organs across sites. Different letters indicate differences among sites according to Tukey’s test (*p* < 0.05).

Site	Sterols	Terpenes	Apiol
Tabarka	1.39 ^a^ ± 1.70	0.29 ^a^ ± 0.34	9.44 ^a^ ± 8.10
Bizerte	1.62 ^a^ ± 2.03	0.50 ^a^ ± 0.46	7.99 ^a^ ± 4.05
Cap Negro	1.79 ^a^ ± 2.28	0.14 ^a^ ± 0.12	5.85 ^a^ ± 2.21
Haouaria	1.12 ^a^ ± 1.33	0.49 ^a^ ± 0.62	6.78 ^a^ ± 8.04
Monastir	1.00 ^a^ ± 1.16	0.79 ^a^ ± 1.05	17.42 ^a^ ± 0.45

## Data Availability

The research data used to support the findings of this study are available from the corresponding author upon request.

## Supplementary Materials

The following supporting information can be downloaded at: https://www.mdpi.com/article/10.3390/plants14213403/s1, Figure S1: Representative stereomicroscope images illustrating confirmed insect herbivory symptoms on *Crithmum maritimum* L.
