# Supplementary material for: Defense and Adaptive Strategies of Crithmum maritimum L. Against Insect Herbivory: Evidence of Phenotypic Plasticity
Source: Plants (Basel). 2025 Nov 6;14(21):3403. doi: 10.3390/plants14213403 (PMC12610603; doi:10.3390/plants14213403)
Supplement: Supplementary file 1 [file plants-14-03403-s001.zip › plants-3892993-supplementary.pdf]

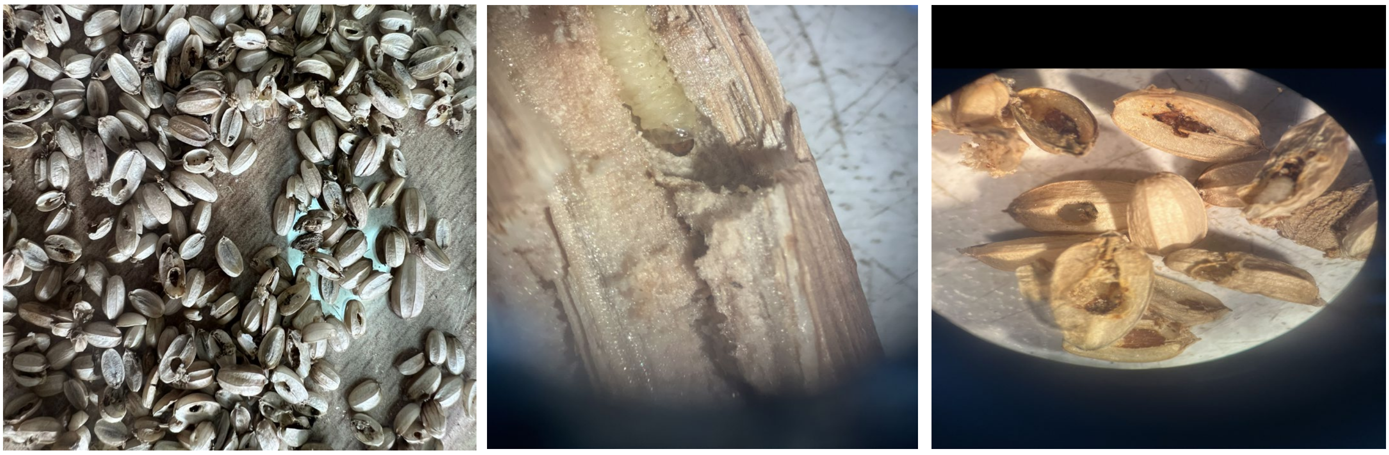

Figure S1. Representative stereomicroscope images illustrating confirmed insect herbivory symptoms on *Crithmum maritimum* L.
